# Temporal dynamics of somatosensory gating and facilitation in the primary somatosensory cortex during finger movements

**DOI:** 10.1093/cercor/bhaf300

**Published:** 2025-11-04

**Authors:** Toshiaki Wasaka, Tetsuo Kida

**Affiliations:** Department of Engineering, Nagoya Institute of Technology, Gokiso, Showa, Nagoya, Aichi 466-8555, Japan; Department of Integrative Physiology, National Institute for Physiological Sciences, 38 Nishigonaka, Myodaiji, Okazaki, Aichi 444-8585, Japan; Department of Integrative Physiology, National Institute for Physiological Sciences, 38 Nishigonaka, Myodaiji, Okazaki, Aichi 444-8585, Japan; Higher Brain Function Unit, Department of Neuronal Information, Institute for Developmental Research, Aichi Developmental Disability Center, 713-8 Kagiya, Kasugai, Aichi 480-0392, Japan; Section of Brain Function Information, Supportive Center for Brain Research, National Institute for Physiological Sciences, 38 Nishigonaka, Myodaiji, Okazaki, Aichi 444-8585, Japan

**Keywords:** dexterity, gating, manual movement, somatosensory evoked magnetic fields

## Abstract

Dexterous manual movements require accurate sensorimotor integration; however, understanding how the primary somatosensory cortex (SI) dynamically processes incoming afferent information during such tasks remains limited. Using magnetoencephalography, we investigated somatosensory evoked magnetic fields during two types of finger movement, varying in speed and force exertion. We focused on three SI components—M20, M30, and M38—occurring within 20 to 40 ms post-stimulation. Across all movement conditions, the M20 and M30 amplitudes were significantly reduced when compared with the stationary condition, reflecting sensory gating, whereas M38 was significantly enhanced during both the rotation and pinch tasks. Further analyses revealed that the reduction in M20 was sensitive to movement speed, and that of M30 was influenced by both speed and force. In contrast, the enhancement of M38 was modulated by the finger movement type. These findings suggest that SI activity is not uniformly inhibited during movement, but selectively modulated in a context-dependent manner. The initial components in the SI may reflect the filtering of predictable inputs, whereas M38 could represent the more complex integration associated with skillful finger movement. Thus, our results suggest a dynamic somatosensory processing mechanism that underpins fine motor control.

## Introduction

Skilled motor activities such as playing musical instruments or performing precise manual tasks are hallmarks of human motor dexterity. These motor activities require continuous interactions between sensory and motor information to form precise feedback loops in the central nervous system. Investigating the cortical processing of somatosensory information during movement is useful for elucidating the mechanisms of motor control, and understanding motor learning and skill acquisition (Wasaka and Kakigi 2012; Ohashi et al. 2019; Akaiwa et al. 2023). Somatosensory evoked potentials (SEPs) and fields (SEFs) are critical for evaluating how sensory information is processed in the brain, and evaluation of the SEPs/SEFs modulation during movement is useful for elucidating the neural mechanisms underlying movement control. Previous studies have shown that the amplitude of short-latency SEPs/SEFs components in the primary somatosensory cortex (SI) decrease during movement execution—this phenomenon is called “gating” (Rushton et al. 1981; Starr and Cohen 1985; Cheron and Borenstein 1987; Tapia et al. 1987). This modulation of the SEPs/SEFs amplitude can occur in two possible ways: (i) occlusion between the somatosensory afferents following electrical stimulation and the afferent signals from the moving muscles, joints, and skin (centripetal gating) and (ii) inhibitory effects of efferent signals from movement-related areas on the somatosensory system (centrifugal gating) (Kakigi and Jones 1986; Jones et al. 1989; Nakata et al. 2003; Wasaka et al. 2003). The functional role of this inhibition of somatosensory information processing is thought to act as a filter mechanism that eliminates sensory information unrelated to motor execution and extracts only the information that is important for efficient motor control. However, activity in the SI during movement is not simply suppressed—instead, it changes depending on the context of the motor task. Somatosensory feedback, originating from cutaneous and proprioceptive receptors, provides essential information for the on-line regulation of movement. The degree of inhibition of SEPs/SEFs during motor execution can be modulated according to the task-dependent requirement for this feedback. For instance, tasks requiring precise coordination of body movement tend to reduce inhibition, reflecting a greater dependence on somatosensory input. (Staines et al. 1997a; Nelson et al. 2001). These results indicate the existence of a neural mechanism that effectively regulates the inhibition of somatosensory information.

Recently, we discovered a short-latency SEF component in which the amplitude increased rather than decreased during dexterous manual movements (Wasaka et al. 2017, 2021). In the task of rotating two balls on the palm using the fingers, the first cortical component appearing at approximately 20 and 30 ms decreased, whereas the subsequent component appearing at approximately 40 ms significantly increased. These findings indicate that information processing in this particular somatosensory area might be enhanced to meet the sensory requirements of precise finger movements. However, the dynamics of the SEP/SEF components during dexterous motor tasks are not fully understood. Neural activity in the SI during movement is strongly influenced by the motor system, and SEP/SEF amplitudes fluctuate depending on the speed of movement and amount of muscle force exerted (Rushton et al. 1981; Cohen and Starr 1985; Rauch et al. 1985; Tinazzi et al. 1998; Sugawara et al. 2016). Clarifying how somatosensory responses are differentially modulated by movement parameters is essential for advancing our understanding of the neural substrates of fine motor control. Therefore, this study aimed to investigate the modulation of SI activation during manual movement, with a focus on the 20 to 40 ms post-stimulation window.

## Materials and methods

### Participants

Twelve healthy, right-handed male volunteers (mean age 26.3 ± 8.1 yr) participated in this study. All participants were in good health and were not taking any medications prior to the experiment. Informed consent was obtained from all participants before the experiment, which was approved by the Ethics Committee of the Nagoya Institute of Technology (approval no. 2020-025).

### Magnetoencephalography acquisitions

Brain activity was measured using a 306-channel magnetoencephalography (MEG) system (Vectorview; Elekta Neuromag Oy, Helsinki, Finland), which comprises 102 identical triple-sensor elements. Each sensor element at a given recording position incorporated one magnetometer and two orthogonal planar-type gradiometers designed to detect neuromagnetic signal variations along the latitudinal and longitudinal directions. In a planar gradiometer, the sensor just over the local cerebral source in the brain detected the maximal magnetic signal. Continuous MEG data were acquired using a bandpass filter of 0.03 to 300 Hz and digitized at a sampling rate of 1,000 Hz.

### Motor tasks

**Fig. 1:**
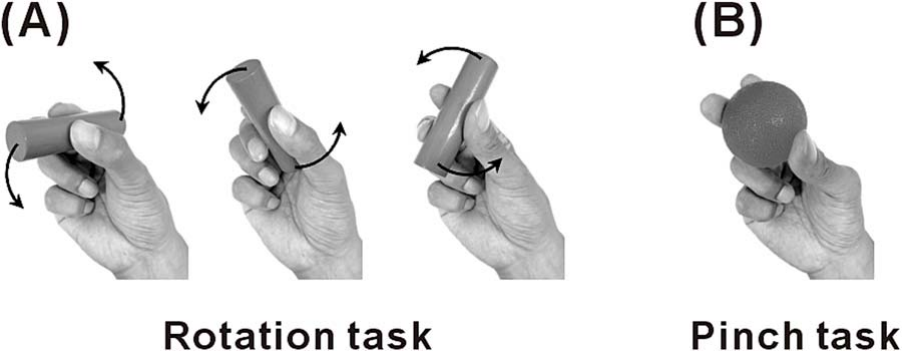
The four motor conditions. Participants performed two kinds of four right-hand finger tasks using the thumb, index, and middle fingers. A) The rotation task consisted of two conditions: one in which the wooden cylinder was rotated continuously at a comfortable pace (normal rotation condition), and the other wherein it was rotated at approximately half the normal speed (slow rotation condition). B) The pinch task consisted of two conditions: one in which a soft silicone ball was pinched intermittently (weak pinch condition), and the other wherein a hard silicone ball was pinched intermittently (strong pinch condition). Throughout each condition, the right median nerve at the wrist was electrically stimulated at motor threshold.

Participants were seated on a chair in a shielded room for MEG measurements. They performed four motor tasks with different contraction intensities and complex finger movements at different movement speeds (Fig. 1). The four motor tasks were as follows: (i) a normal rotation task, involving continuous rotation of a wooden cylinder (diameter, 20 mm; length, 80 mm; weight, 22 g) at a comfortable pace; (ii) a slow rotation task, involving continuous rotation of the same cylinder at half the speed of the normal rotation task; (iii) a weak pinch task: pinching a soft silicone ball (diameter, 53 mm; weight, 60 g; 5 lb of force required to deform 20% of its diameter); (iv) a strong pinch task, involving pinching a hard silicone ball (diameter, 53 mm; weight, 62 g; 10 lb of force required to deform 20% of its diameter). The instruction for the two pinch tasks was to pinch the ball for approximately 10 s and then relax for approximately 2 to 3 s. The subjects then repeated this sequence of movements. The number of rotations was counted from 20 s recording during the rotation tasks. All motor tasks used only the thumb, index finger, and middle finger of the right hand. The duration of each motor task was approximately 3 min.

### Stimulation for SEF recording

The right median nerve was electrically stimulated on the palmar side of the wrist, with the cathode placed 2 cm proximal to the anode (NM-422B; Nihon Kohden, Tokyo, Japan). Constant current square wave pulses with a duration of 0.2 ms were delivered with an interstimulus interval of 500 ms. The stimulus intensity was set at the motor threshold (10.0 ± 1.8 mA), eliciting a slight twitch in the abductor pollicis brevis muscle. The experimenter monitored that the current value did not change during the presentation of electrical stimulation.

### SEF measurements and data analysis

The participants were instructed to concentrate on their manual movements during the four motor tasks and not pay attention to continuous electrical stimulation. As a control condition, we also recorded SEFs during the stationary state (stationary condition). In this condition, participants were instructed to position their hands supine on the table and remain relaxed. The order of the four motor tasks and the stationary condition were randomized among the subjects. In each condition, we averaged 300 stimuli to obtain the SEF waveforms. The analysis time was 300 ms, including a 100-ms prestimulus period. The data recorded during the 100 ms preceding the stimulus onset were used for the baseline calculations.

The equivalent current dipoles (ECDs) in the SEF components were modeled using the time-varying current dipole method (Hämäläinen et al. 1993). In the central hemisphere contralateral to the stimulus side, the earliest deflection of the SEF waveform was observed at approximately 20 ms in all subjects. The ECD that best explained the main deflection was determined using a least-squares search based on a selection of 16 to 18 channels. The goodness-of-fit (GOF) value was calculated to indicate the extent to which the ECD accounted for the measured variance in the SEF component. Only the ECD, which accounted for more than 80% of the GOF, was accepted. Subsequently, a time-varying dipole model was constructed using all MEG channels, allowing the strengths of the identified ECDs to vary throughout the entire period, while maintaining ECD locations and orientations.

The moments of the ECDs showed three distinct peak components in all participants. The first deflection reached its peak at approximately 20 ms (M20), followed by the second largest component at approximately 30 ms (M30). The final component exhibited a smaller deflection, peaking at approximately 38 ms (M38).

### Statistical analysis

To examine SI changes that occurred with finger movement, the amplitudes of the three ECD components were compared among the stationary condition and the four motor tasks using one-way repeated measures analysis of variance (ANOVA), using the condition (normal rotation, slow rotation, weak pinch and strong pinch tasks, stationary condition) as a factor. To analyze the assumption of sphericity prior to repeated ANOVA, we used Mauchly’s test of sphericity. To compare each motor task with the stationary condition, a post hoc analysis was performed by Dunnett’s test.

In addition, to examine the relationship between different types of finger movements and the level of muscle activity in sensorimotor integration, we performed two-way repeated measures ANOVA. The analysis included the movement type (rotation, pinch) and amount of muscle activity (small, large) as factors. The normal rotation and strong pinch tasks involved a larger extent of muscle activity, whereas the slow rotation and weak pinch tasks involved a smaller extent of muscle activity. Post hoc analysis was then performed by the paired t-test. In addition, we calculated the effect sizes for results of ANOVA (partial eta-squared, η^2^) and paired t-test (Cohen’s d). The level of statistical significance was set at 5% (*P* < 0.05).

## Results

### Motor tasks

The rotation of the wooden cylinder task had two conditions with different manual movement speeds. The results showed that the normal rotation task was performed 18.7 ± 5.9 rpm, while the slow rotation task was performed 8.8 ± 2.7 rpm. The subjects were instructed to perform the slow rotation task at half the speed of the normal rotation task, and they performed the tasks as instructed.

### Modulation in the SI activities during manual movement

**Fig. 2:**
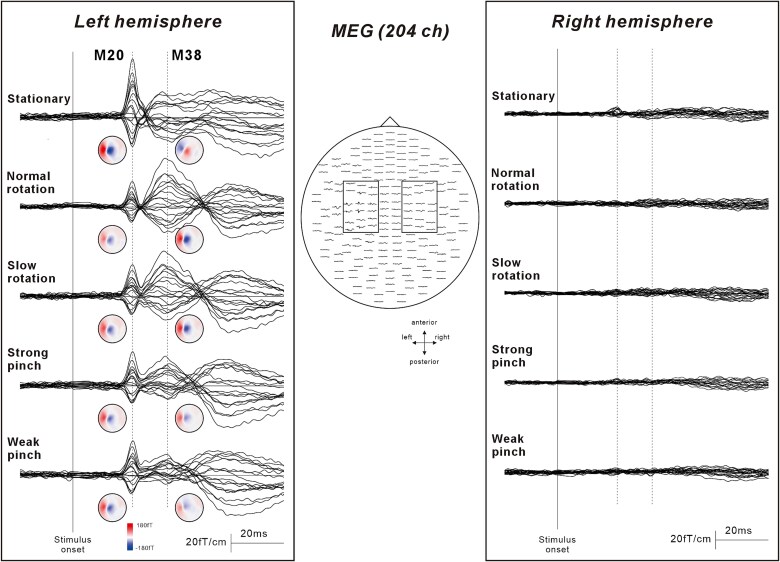
The somatosensory evoked magnetic field (SEF) waveforms following stimulation of the right median nerve. Middle figure indicates diagram of 204 planar-gradiometer location viewed from the top of the head. Right and left square indicate the sensor location around the primary somatosensory cortex in both hemispheres. Enlarged right and left waveforms indicate the grand-averaged superimposed SEF waveforms around the primary somatosensory cortex for five experimental conditions: Stationary, Normal rotation, Slow rotation, Strong pinch, and Weak pinch. Following right median nerve stimulation, clear deflections are observed over the central region contralateral to stimulation (left hemisphere). The small circular maps below the waveforms are topographical maps of magnetic field at the latencies of M20 and M38. In the four motor conditions, M20 shows a decrease, whereas M38 demonstrates an increase compared to the stationary condition. Additionally, the polarity of the magnetic field pattern of M38 is reversed between the stationary and four movement conditions, indicating a change in the direction of the magnetic flow associated with finger movement. In contrast, no deflection is observed in the central region ipsilateral to stimulation.

Figure 2 shows the grand-averaged SEF waveforms following stimulation of the right median nerve under the five experimental conditions. Clear components were obtained from the gradiometers placed at the central region in the hemisphere contralateral to the stimulated side (left hemisphere) at a latency of less than 40 ms. In contrast, no deflection was observed around the central region ipsilateral to stimulation.

**Fig. 3:**
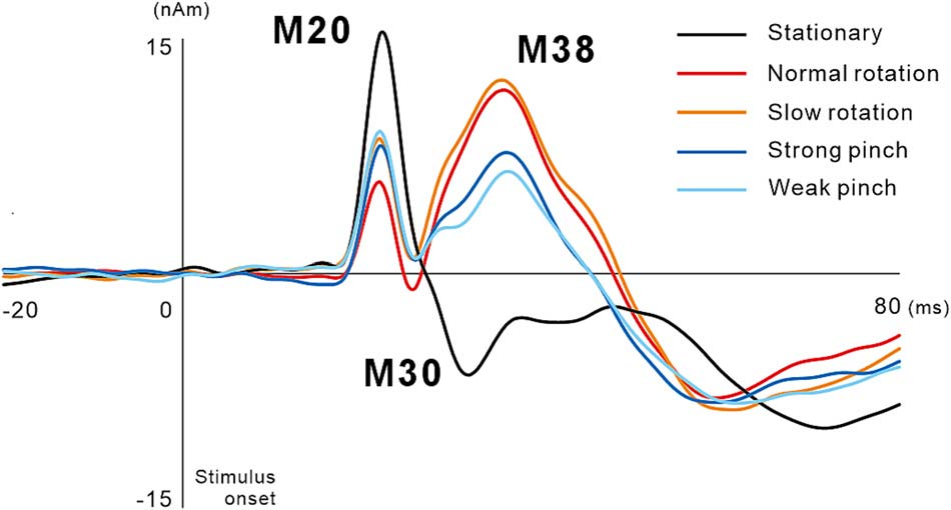
Temporal changes of the grand-averaged ECD waveforms of the primary somatosensory cortex in 12 participants. While the peak amplitude of M20 and M30 in the four finger movement tasks decreased when compared with those in the stationary condition, the peak amplitude of M38 increased in the pinch and rotation tasks.

Figure 3 shows the grand-averaged ECD waveforms for the stationary condition and the four motor tasks. Three components were identified, with the first peaking at approximately 20 ms following stimulation (M20), the second peaking at approximately 30 ms (M30), and the third component showing a small peak at approximately 40 ms (M38).

Table 1 shows the results of one-way repeated measures ANOVA for each component to examine the differences among the four motor tasks and the stationary condition. Significant main effects were found for the peak amplitude of the ECD moment for M20 (F_(4, 44)_ = 17.09, *P* < 0.01, η^2^ = 0.61), M30 (F_(4, 44)_ = 8.34, *P* < 0.01, η^2^ = 0.43, ε = 0.31), and M38 (F_(4, 44)_ = 19.80, *P* < 0.01, η^2^ = 0.64, ε = 0.64). Post hoc analyses showed that the ECD moments for M20 and M30 were significantly smaller in the four motor tasks than in the stationary condition (all *P* < 0.01). In contrast, the ECD moment for M38 was significantly larger in the four motor tasks than in the stationary condition (all *P* < 0.01).

**Table 1:** Peak amplitude and latency of three components of the ECD moment in the stationary and four motor tasks.

	Stationary	Rotation task		Pinch task		η^2^
		Normal	Slow	Strong	Weak	
Amplitude (nAm)						
M20	17.8 (7.1)	7.7 (5.5)^**^	10.6 (6.0)^**^	10.0 (4.6)^**^	10.6 (6.1)^**^	0.61
M30	−7.1 (8.2)	−1.1 (2.7)^**^	0.8 (3.2)^**^	−0.1 (1.8)^**^	−1.1 (2.6)^**^	0.43
M38	0.4 (6.3)	13.7 (9.4)^**^	14.0 (9.6)^**^	8.5 (4.8)^**^	6.9 (6.3)^**^	0.64
Latency (ms)						
M20	21.6 (0.8)	21.3 (0.9)	21.5 (0.9)	21.5 (0.9)	21.7 (1.0)	0.10
M30	28.6 (3.7)	25.6 (1.0)^*^	25.8 (1.0)^*^	27.1 (3.0)^*^	27.6 (2.8)^*^	0.33
M38	37.0 (5.7)	35.7 (4.0)	35.0 (3.8)	35.6 (3.4)	35.8 (4.4)	0.15

Data are expressed as the mean (standard deviation).

^*^ *P* < 0.05, ^**^P < 0.01 (compared with the stationary condition).

### Different types of movement and amount of muscle activity

**Fig. 4:**
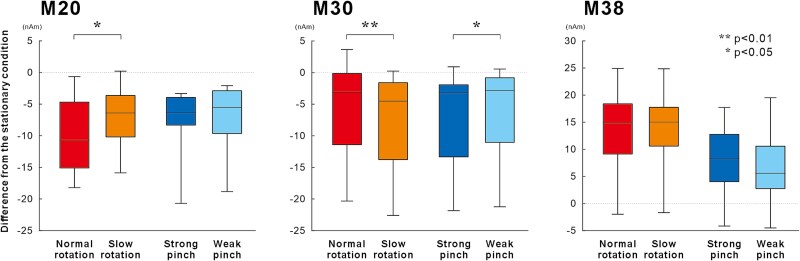
Mean amplitude of the three ECD components of the primary somatosensory cortex during the four motor tasks. The data represent the changes from the stationary condition for each motor task. M20 reduction is larger for the normal rotation task compared to the slow rotation task (*P* < 0.05), with no difference between the strong and weak pinch tasks. Additionally, the M30 inhibition is larger for the slow rotation task than that for the normal rotation task (*P* < 0.01) and smaller for the weak pinch task than for the strong pinch task (*P* < 0.05).

To assess variations due to differences in the type of movement and amount of muscle activity, we calculated the change from the stationary condition for each motor task. Figure 4 shows the results of two-way ANOVA to investigate which factor of finger movement influenced the early components of the SEF, with movement type and amount of muscle activity as factors. Significant main effect was found for the amplitude of the ECD moment for M20 in the amount of muscle activity (F_(1, 11)_ = 6.66, *P* < 0.05, η^2^ = 0.38) and that of M38 in the movement type (F_(1,11)_ = 14.83, *P* < 0.01, η^2^ = 0.57). In addition, significant interactions between the movement type and amount of muscle activity were found for the ECD moments for M20 (F_(1, 11)_ = 6.42, *P* < 0.05, η^2^ = 0.37) and M30 (F_(1, 11)_ = 21.13, *P* < 0.01, η^2^ = 0.66). Post hoc tests showed that the amplitude of M20 was significantly smaller for the normal rotation task than for the slow rotation task (*P* < 0.05, Cohen’s d = 0.84), whereas no significant differences were observed between the strong pinch and weak pinch tasks. Furthermore, the reduction in the amplitude of M30 was significantly larger for the slow rotation task than for the normal rotation task (*P* < 0.01, Cohen’s d = 0.95), whereas that for the weak pinch task was significantly smaller than that for the strong pinch task (*P* < 0.05, Cohen’s d = 0.69).

## Discussion

Manual movements, which are essential for exploring and interacting with the environment, require precise somatosensory feedback. In this study, we investigated changes in the processing of somatosensory information during a variety of finger movements. The results showed that the short-latency responses of the SI varied specifically with the type of finger movement, speed of movement, and amount of muscle exertion. The M20 amplitude was strongly influenced by the movement speed in the rotation task, while the M30 amplitude was strongly influenced by speed in the rotation task and the amount of muscle exertion in the pinch task. In contrast, M38 showed differences in the type of finger movement, but no change in the amount of motor activity. Given that afferent information is generated from muscle activity induced by electrical stimulation, somatosensory input from such stimulation likely interferes at the level of the SI. The short-latency components of M20 and M30 appear unaffected by this centripetal effect, whereas M38 is potentially influenced due to its relatively slower latency. However, since the interference of sensory information acts to suppress SEF amplitude, the increase in M38 is hypothesized to result from enhanced neural activity that overcomes this inhibitory effect. Thus, our results show that the distinctive processing of somatosensory information underlies manual dexterity.

### Reduction of the SEP/SEF components during manual movement

Examining SEP/SEF amplitudes during movement provides clues for the elucidation of the function of somatosensory information in motor control. The reduction of short-latency SEP/SEF components during movement execution, known as sensory gating, has long been consistently observed and is generally accepted in this field (Papakostopoulos et al. 1975; Rushton et al. 1981; Jones et al. 1989). This occurs through centrifugal gating from motor areas that inhibit the SI and centripetal gating from interference between movement-related and electrically stimulated somatosensory information. Therefore, these two neural processes are believed to function by suppressing the activity of the somatosensory area to eliminate somatosensory information that is not required for movement.

Although it is consistent with reduced activation in the SI during movement, there is an ongoing debate regarding the influence of muscle movement on the amplitude of the short-latency SEP/SEF components. For example, some previous studies have shown no change in the cortical component around 20 ms during movement (Cheron and Borenstein 1987, 1991; Cohen and Starr 1987; Tapia et al. 1987; Kirimoto et al. 2014; Sugawara et al. 2016; Takahara et al. 2020), while others have found a reduction in this component during movement (Abbruzzese et al. 1981; Jones 1981; Seyal et al. 1987; Huttunen and Hömberg 1991; Hoshiyama and Kakigi 1999; Lei et al. 2018). Our group reported that this component did not change during the preparatory period (Wasaka et al. 2003, 2005a; Kida et al. 2006a, 2006b, 2006c); instead, a significant reduction was observed in the motor task of using fingers to manipulate objects (Wasaka et al. 2017, 2021). The present study showed that the M20 amplitude decreased during motor tasks involving pinching and rotating objects using the fingertips. Generally, the initial stage of brain processing occurs at approximately 20 ms in area 3b of the somatosensory cortex (Allison et al. 1991; Hirata et al. 2024), which receives information from skin receptors, particularly from the fingertips (Iwamura et al. 1993). Therefore, the substantial somatosensory information generated by movement might contribute to the reduction in M20 through the centripetal mechanism.

The subsequent component, which appears at approximately 30 ms, is strongly influenced by muscle contraction and has consistently been reported to decrease in amplitude (Cheron and Borenstein 1987; Seyal et al. 1987; Hoshiyama and Sheean 1998; Sugawara et al. 2016). Previously, we consistently observed a reduction in M30 during finger movement (Wasaka et al. 2012, 2017, 2021; Wasaka and Kakigi 2012), demonstrating that this component is strongly affected by movement. The precise origin of M30 is uncertain, but it may originate from area 4 of the primary motor cortex (Kawamura et al. 1996) or area 3b of the somatosensory cortex (Kakigi 1994). The differing responses of M20 and M30 to variations in stimulus intensity (Lin et al. 2003) and timing (Wikström et al. 1996) suggest that distinct brain regions may be responsible for the changes during voluntary movement.

### Facilitation of the SEP/SEF components during manual movement

During manual movement, activity in the SI is inhibited, followed by facilitation of complex finger movements. Previously, we showed that the early components of SEFs are diminished in amplitude during manual movement, whereas the subsequent smaller component, M38, exhibits a significant increase when movements involve the manipulation of objects using the fingertips (Wasaka et al. 2017, 2021). Although it is assumed that the brain has mechanisms to regulate sensory signals from moving body parts to prevent unnecessary reflexes and muscle actions, these results imply that the modification of somatosensory information processing with movement may be more complicated than that with conventional simple suppression.

The inhibition of short-latency SEP gating is influenced by the kinesthetic demands required for controlling movement (Staines et al. 1997a; Legon and Staines 2006). However, this mechanism regulates the amount of short-latency SEF inhibition, which is unlikely to explain the observed enhancement of M38. In contrast, previous studies have shown that the SI component is enhanced when objects are explored manually (Huttunen and Hömberg 1991; Knecht et al. 1993). Efficient object manipulation requires precise control of finger forces and movements, suggesting that somatosensory feedback from the fingers plays a crucial role in facilitating M38 responses during such tasks.

Signals from the muscle spindles and cutaneous receptors provide essential feedback for controlling movement; however, these are processed differently in the SI. Previously, Confais et al. (2017) demonstrated that proprioceptive information processing in the SI was enhanced during voluntary movement, whereas the processing of cutaneous information was inhibited. In the present study, we stimulated the median nerve, which carries afferent inputs from both cutaneous and proprioceptive receptors, for SEF recording. Therefore, we were unable to determine which type of somatosensory input specifically contributed to the enhancement of the M38.

The anatomical sources of the SEP/SEF components in the range of 20 to 40 ms involve a temporal sequence of activity in Brodmann’s area 3b, 4, 1, and the posterior parietal cortex (Inui et al. 2004). Since intracranial SEP recordings in humans have previously shown that the P25–N35 component is generated in the anterior crown of the postcentral gyrus in area 1 (Allison et al. 1989), we assumed that the activity in these regions is responsible for the appearance of the M38 component. The hierarchical information processing of the somatosensory system has a small receptive field in area 3b but complex processing across many fingers in areas 1 and 2 (Iwamura 1998). Notably, previous experiments using muscimol to inactivate area 2 reported disruption of manual coordination of grasping objects using the fingertips (Hikosaka et al. 1985). Since the motor task in this study involved cooperative movement of the thumb, index, and middle fingers, we believe that integrating somatosensory information from multiple fingers leads to an enhancement of the M38 amplitude.

### Changes in muscle activity parameters and neuronal activities in the SI

A systematic examination of SEF component variability under diverse motor conditions is essential for elucidating sensorimotor mechanisms that support the integration of afferent feedback and motor output during skilled manual actions. Variations in the SEP/SEF amplitudes during movement depend on the type and parameters of muscle activity. Since different types of finger movement show varying activities in the sensorimotor areas (Ehrsson et al. 2000; Lei et al. 2018), we expected changes in the SEF components. However, although M38 revealed a significant main effect in the type of movement, no differences were observed in M20 and M30. These findings suggest that information processing of the short-latency SEF components performed distinct sensorimotor integration based on the specific features of the finger motor task. Although the amplitude of M38 previously increased with fingertip movement (Wasaka et al. 2017, 2021), its relationship with movement parameters has not been investigated. Therefore, further research is required to clarify how this component changes with the manual task.

Higher strengths and speeds of muscle contraction lead to larger amounts of neural activities in sensorimotor areas, resulting in greater reduction of the SEP/SEF amplitudes by the centrifugal and centripetal gating effects. Previous studies showed that the gating of SEPs was influenced by the degree of contractile force (Cohen and Starr 1985; Tinazzi et al. 1998; Sakamoto et al. 2004; Wasaka et al. 2005b; Sugawara et al. 2016; Lei et al. 2018) and the speed of contraction (Rushton et al. 1981; Angel and Malenka 1982; Rauch et al. 1985; Staines et al. 1997b). In this study, we found that the M20 amplitude showed a significant reduction during both rotation and pinch tasks when a higher level of muscle activities was applied. Since higher intensity force exertion and speeds of muscle contraction result in larger activation in the motor areas (Slobounov et al. 2004; Wasaka et al. 2005b), the change in M20 resulting from the difference in muscle activity levels might have been prompted by inhibition from the motor areas to the SI. In contrast, the modulation of M30 demonstrated a significant reduction during the slow rotation task when compared to the normal rotation task. We expected that a faster speed of movement would lead to a larger reduction in SEF amplitude; however, this finding indicated that the inhibition in M30 was more pronounced during the slow rotation movements. This may be explained by the fact that cutaneous mechanoreceptors in the fingers exhibit dynamic responses depending on the cycle of movement, and the response activity of slow adapting receptors increases as the movement frequency decreases (Hulliger et al. 1979). Since the rotation task was performed while holding the object without dropping it, a greater amount of afferent information from the fingertips was generated during slower movements. Consequently, it is likely that the centripetal mechanism contributes to a decrease in the M30 amplitude. Alternatively, the centrifugal gating effect may also contribute to the larger inhibition observed in M30 during the slow rotation task. In addition to the centrifugal gating effect from motor cortical areas, prefrontal cortex contributions may also play an important role in modulating somatosensory processing. Brown et al. (2015) demonstrated that early SEP components were selectively enhanced depending on the relevance of somatosensory information for performing a motor task, and that this facilitation was abolished following inhibition of the dorsolateral prefrontal cortex (DLPF) induced by continuous theta burst stimulation. Because the DLPF is involved in controlling the processing of task-related sensory information (Knight et al. 1999), we hypothesized that prefrontal networks filtering relevant sensory inputs influence the complex pattern of short-latency SEF modulation associated with differences in motor tasks and muscle activity. Furthermore, the specific relevance of somatosensory information in a motor task can selectively affect early SEP components originating from proprioceptive or cutaneous sources (Staines et al. 2000). Although the present motor task did not control the relevance of somatosensory information from cutaneous and muscle receptors, the relevance of such somatosensory information may have varied depending on the motor tasks. These results highlight the intricate relationship between afferent feedback and motor output, suggesting that both centrifugal and centripetal mechanisms dynamically modulate SEF components, depending on the specific demands of the manual task.

### Research limitations

We have reported novel findings on the processing of somatosensory information during finger movements; however, several limitations should be noted. First, the source localization of the M38 component could not be reliably estimated due to its small amplitude in the stationary condition, rendering our anatomical interpretation of short-latency components somewhat speculative. Second, electrical stimulation was delivered continuously at 2 Hz without controlling for the movement phase, preventing precise clarification of the relationship between stimulus timing and motor execution. Although the rotation task involved movements of three fingers, the movement of each finger was not recorded, leaving the exact timing of stimulation uncertain. In the pinch tasks, participants were instructed to alternate between contraction and relaxation phases to minimize fatigue, thereby ensuring that both dynamic and static phases were included. Nevertheless, because no behavioral recordings were obtained, we could not confirm whether the distribution of movement phase was consistent across all tasks. Thus, variations in the timing of dynamic and static stimulation timing may have partially influenced the SEF modulation. Third, attentional demands and task difficulty may also have influenced the SEF components; however, these factors were not systematically controlled. Fourth, muscle activity and precise pinch force were not recorded, and thus muscle exertion across conditions could only be inferred from task instructions and object properties, limiting interpretations related to force-dependent modulation. Although stimulation intensity was carefully monitored throughout the experiment, the inability to confirm stable M-wave amplitude during movement tasks further limits our ability to directly verify that peripheral activation remained constant across tasks. Demonstrating stable M-wave constancy would have allowed stronger claims regarding the specific modulation of motor tasks in SEF amplitude. Finally, to obtain sufficiently clear SEF waveforms, we adopted the stimulation intensity at the motor threshold, which inevitably induced slight muscle twitches. While it is unlikely that reafferent feedback from these twitches accounts for the observed effects—since the same stimulation intensity was applied in both stationary and movement conditions—we cannot completely exclude this possibility, particularly for the later M38 component. Future study using stimulus intensity below the motor threshold is warranted to eliminate the contribution of reafferent somatosensory information and to clarify the mechanisms underlying sensory gating and facilitation.

## Conclusion

Our study demonstrated that somatosensory cortical activities were modulated in a task-specific manner during dexterous manual movements. While the early cortical SEF components—M20 and M30—were suppressed during movement, consistent with traditional gating mechanisms, the subsequent M38 component was enhanced during skilled object manipulation. Thus, our study suggested that the somatosensory system does not simply filter afferent inputs. These findings highlight the existence of context-dependent somatosensory processing systems in the SI. However, future studies are required to clarify the neural mechanisms underlying dexterous manual movement.

## Data Availability

The data are available from the corresponding author upon reasonable request.

## References

[ref1] Abbruzzese G, Ratto S, Favale E, Abbruzzese M. 1981. Proprioceptive modulation of somatosensory evoked potentials during active or passive finger movements in man. J Neurol Neurosurg Psychiatry. 44:942–949. 10.1136/jnnp.44.10.942. 7310412 PMC491182

[ref2] Akaiwa M et al. 2023. Effects of repetitive practice of motor tasks on somatosensory gating. Front Hum Neurosci. 17:1178509. 10.3389/fnhum.2023.1178509. 37063102 PMC10090363

[ref3] Allison T et al. 1989. Human cortical potentials evoked by stimulation of the median nerve. I. Cytoarchitectonic areas generating short-latency activity. J Neurophysiol. 62:694–710. 10.1152/jn.1989.62.3.694. 2769354

[ref4] Allison T, McCarthy G, Wood CC, Jones SJ. 1991. Potentials evoked in human and monkey cerebral cortex by stimulation of the median nerve. A review of scalp and intracranial recordings. Brain. 114 (Pt 6):2465–2503. 10.1093/brain/114.6.2465. 1782527

[ref5] Angel RW, Malenka RC. 1982. Velocity-dependent suppression of cutaneous sensitivity during movement. Exp Neurol. 77:266–274. 10.1016/0014-4886(82)90244-8. 7095061

[ref6] Brown KE, Ferris JK, Amanian MA, Staines WR, Boyd LA. 2015. Task-relevancy effects on movement-related gating are modulated by continuous theta-burst stimulation of the dorsolateral prefrontal cortex and primary somatosensory cortex. Exp Brain Res. 233:927–936. 10.1007/s00221-014-4168-6. 25511167

[ref7] Cheron G, Borenstein S. 1987. Specific gating of the early somatosensory evoked potentials during active movement. Electroencephalogr Clin Neurophysiol. 67:537–548. 10.1016/0013-4694(87)90056-3. 2445546

[ref8] Cheron G, Borenstein S. 1991. Gating of the early components of the frontal and parietal somatosensory evoked potentials in different sensory-motor interference modalities. Electroencephalogr Clin Neurophysiol. 80:522–530. 10.1016/0168-5597(91)90134-j. 1720728

[ref9] Cohen LG, Starr A. 1985. Vibration and muscle contraction affect somatosensory evoked potentials. Neurology. 35:691–698. 10.1212/wnl.35.5.691. 3157885

[ref10] Cohen LG, Starr A. 1987. Localization, timing and specificity of gating of somatosensory evoked potentials during active movement in man. Brain. 110 ( Pt 2):451–467. 10.1093/brain/110.2.451. 3567532

[ref11] Confais J, Kim G, Tomatsu S, Takei T, Seki K. 2017. Nerve-specific input modulation to spinal neurons during a motor task in the monkey. J Neurosci. 37:2612–2626. 10.1523/JNEUROSCI.2561-16.2017. 28159911 PMC6596638

[ref12] Ehrsson HH et al. 2000. Cortical activity in precision- versus power-grip tasks: an fMRI study. J Neurophysiol. 83:528–536. 10.1152/jn.2000.83.1.528. 10634893

[ref13] Hämäläinen M, Hari R, Ilmoniemi RJ, Kunuutila J, Lounasmaa OV. 1993. Magnetoencephalography—theory, instrumentation, and application to noninvasive studies of the working human brain. Rev Mod Phys. 65:413–497. 10.1103/RevModPhys.65.413.

[ref14] Hikosaka O, Tanaka M, Sakamoto M, Iwamura Y. 1985. Deficits in manipulative behaviors induced by local injections of muscimol in the first somatosensory cortex of the conscious monkey. Brain Res. 325:375–380. 10.1016/0006-8993(85)90344-0. 3978429

[ref15] Hirata A et al. 2024. High-resolution EEG source localization in personalized segmentation-free head model with multi-dipole fitting. Phys Med Biol. 69:055013. 10.1088/1361-6560/ad25c3. 38306964

[ref16] Hoshiyama M, Kakigi R. 1999. Changes of somatosensory evoked potentials during writing with the dominant and non-dominant hands. Brain Res. 833:10–19. 10.1016/s0006-8993(99)01443-2. 10375672

[ref17] Hoshiyama M, Sheean G. 1998. Changes of somatosensory evoked potentials preceding rapid voluntary movement in go/No-go choice reaction time task. Brain Res Cogn Brain Res. 7:137–142. 10.1016/s0926-6410(98)00018-4. 9774718

[ref18] Hulliger M, Nordh E, Thelin AE, Vallbo AB. 1979. The responses of afferent fibres from the glabrous skin of the hand during voluntary finger movements in man. J Physiol. 291:233–249. 10.1113/jphysiol.1979.sp012809. 480210 PMC1280897

[ref19] Huttunen J, Hömberg V. 1991. Modification of cortical somatosensory evoked potentials during tactile exploration and simple active and passive movements. Electroencephalogr Clin Neurophysiol. 81:216–223. 10.1016/0168-5597(91)90075-9. 1710971

[ref20] Inui K, Wang X, Tamura Y, Kaneoke Y, Kakigi R. 2004. Serial processing in the human somatosensory system. Cereb Cortex. 14:851–857. 10.1093/cercor/bhh043. 15054058

[ref21] Iwamura Y . 1998. Hierarchical somatosensory processing. Curr Opin Neurobiol. 8:522–528. 10.1016/s0959-4388(98)80041-x. 9751655

[ref22] Iwamura Y, Tanaka M, Sakamoto M, Hikosaka O. 1993. Rostrocaudal gradients in the neuronal receptive field complexity in the finger region of the alert monkey’s postcentral gyrus. Exp Brain Res. 92:360–368. 10.1007/BF00229023. 8454001

[ref23] Jones SJ . 1981. An “interference” approach to the study of somatosensory evoked potentials in man. Electroencephalogr Clin Neurophysiol. 52:517–530. 10.1016/0013-4694(81)91427-9. 6172252

[ref24] Jones SJ, Halonen JP, Shawkat F. 1989. Centrifugal and centripetal mechanisms involved in the ‘gating’ of cortical SEPs during movement. Electroencephalogr Clin Neurophysiol. 74:36–45. 10.1016/0168-5597(89)90049-x. 2463147

[ref25] Kakigi R . 1994. Somatosensory evoked magnetic fields following median nerve stimulation. Neurosci Res. 20:165–174. 10.1016/0168-0102(94)90034-5. 7808699

[ref26] Kakigi R, Jones SJ. 1986. Influence of concurrent tactile stimulation on somatosensory evoked potentials following posterior tibial nerve stimulation in man. Electroencephalogr Clin Neurophysiol. 65:118–129. 10.1016/0168-5597(86)90044-4. 2419100

[ref27] Kawamura T et al. 1996. Neuromagnetic evidence of pre- and post-central cortical sources of somatosensory evoked responses. Electroencephalogr Clin Neurophysiol. 100:44–50. 10.1016/0168-5597(95)00217-0. 8964262

[ref28] Kida T, Wasaka T, Nakata H, Akatsuka K, Kakigi R. 2006a. Centrifugal regulation of a task-relevant somatosensory signal triggering voluntary movement without a preceding warning signal. Exp Brain Res. 173:733–741. 10.1007/s00221-006-0448-0. 16636794

[ref29] Kida T, Wasaka T, Nakata H, Kakigi R. 2006b. Centrifugal regulation of task-relevant somatosensory signals to trigger a voluntary movement. Exp Brain Res. 169:289–301. 10.1007/s00221-005-0141-8. 16307265

[ref30] Kida T et al. 2006c. Centrifugal regulation of human cortical responses to a task-relevant somatosensory signal triggering voluntary movement. NeuroImage. 32:1355–1364. 10.1016/j.neuroimage.2006.05.015. 16806987

[ref31] Kirimoto H et al. 2014. Sensorimotor modulation differs with load type during constant finger force or position. PLoS One. 9:e108058. 10.1371/journal.pone.0108058. 25233353 PMC4169486

[ref32] Knecht S, Kunesch E, Buchner H, Freund HJ. 1993. Facilitation of somatosensory evoked potentials by exploratory finger movements. Exp Brain Res. 95:330–338. 10.1007/BF00229790. 8224057

[ref33] Knight RT, Staines WR, Swick D, Chao LL. 1999. Prefrontal cortex regulates inhibition and excitation in distributed neural networks. Acta Psychol. 101:159–178. 10.1016/s0001-6918(99)00004-9. 10344184

[ref34] Legon W, Staines WR. 2006. Predictability of the target stimulus for sensory-guided movement modulates early somatosensory cortical potentials. Clin Neurophysiol. 117:1345–1353. 10.1016/j.clinph.2006.02.024. 16644272

[ref35] Lei Y, Ozdemir RA, Perez MA. 2018. Gating of sensory input at subcortical and cortical levels during grasping in humans. J Neurosci. 38:7237–7247. 10.1523/JNEUROSCI.0545-18.2018. 29976624 PMC6096046

[ref36] Lin YY et al. 2003. Differential effects of stimulus intensity on peripheral and neuromagnetic cortical responses to median nerve stimulation. NeuroImage. 20:909–917. 10.1016/S1053-8119(03)00387-2. 14568461

[ref37] Nakata H, Inui K, Wasaka T, Nishihira Y, Kakigi R. 2003. Mechanisms of differences in gating effects on short- and long-latency somatosensory evoked potentials relating to movement. Brain Topogr. 15:211–222. 10.1023/a:1023908707851. 12866825

[ref38] Nelson AJ, Brooke JD, McIlroy WE, Bishop DC, Norrie RG. 2001. The gain of initial somatosensory evoked potentials alters with practice of an accurate motor task. Brain Res. 890:272–279. 10.1016/s0006-8993(00)03136-x. 11164793

[ref39] Ohashi H, Gribble PL, Ostry DJ. 2019. Somatosensory cortical excitability changes precede those in motor cortex during human motor learning. J Neurophysiol. 122:1397–1405. 10.1152/jn.00383.2019. 31390294 PMC6843109

[ref40] Papakostopoulos D, Cooper R, Crow HJ. 1975. Inhibition of cortical evoked potentials and sensation by self-initiated movement in man. Nature. 258:321–324. 10.1038/258321a0. 1196355

[ref41] Rauch R, Angel RW, Boylls CC. 1985. Velocity-dependent suppression of somatosensory evoked potentials during movement. Electroencephalogr Clin Neurophysiol. 62:421–425. 10.1016/0168-5597(85)90051-6. 2415337

[ref42] Rushton DN, Rothwell JC, Craggs MD. 1981. Gating of somatosensory evoked potentials during different kinds of movement in man. Brain. 104:465–491. 10.1093/brain/104.3.465. 7272711

[ref43] Sakamoto M et al. 2004. Load- and cadence-dependent modulation of somatosensory evoked potentials and soleus H-reflexes during active leg pedaling in humans. Brain Res. 1029:272–285. 10.1016/j.brainres.2004.09.054. 15542082

[ref44] Seyal M, Ortstadt JL, Kraft LW, Gabor AJ. 1987. Effect of movement on human spinal and subcortical somatosensory evoked potentials. Neurology. 37:650–655. 10.1212/wnl.37.4.650. 3561777

[ref45] Slobounov S, Hallett M, Newell KM. 2004. Perceived effort in force production as reflected in motor-related cortical potentials. Clin Neurophysiol. 115:2391–2402. 10.1016/j.clinph.2004.05.021. 15351382

[ref46] Staines WR, Brooke JD, Cheng J, Misiaszek JE, MacKay WA. 1997a. Movement-induced gain modulation of somatosensory potentials and soleus H-reflexes evoked from the leg. I. Kinaesthetic task demands. Exp Brain Res. 115:147–155. 10.1007/pl00005674. 9224842

[ref47] Staines WR, Brooke JD, Misiaszek JE, McIlroy WE. 1997b. Movement-induced gain modulation of somatosensory potentials and soleus H-reflexes evoked from the leg. II. Correlation with rate of stretch of extensor muscles of the leg. Exp Brain Res. 115:156–164. 10.1007/pl00005676. 9224843

[ref48] Staines WR, Brooke JD, McIlroy WE. 2000. Task-relevant selective modulation of somatosensory afferent paths from the lower limb. NeuroReport. 11:1713–1719. 10.1097/00001756-200006050-00024. 10852231

[ref49] Starr A, Cohen LG. 1985. ‘Gating’ of somatosensory evoked potentials begins before the onset of voluntary movement in man. Brain Res. 348:183–186. 10.1016/0006-8993(85)90377-4. 4063823

[ref50] Sugawara K et al. 2016. Effect of muscle contraction strength on gating of somatosensory magnetic fields. Exp Brain Res. 234:3389–3398. 10.1007/s00221-016-4736-z. 27435203

[ref51] Takahara T, Yamaguchi H, Seki K, Onodera S. 2020. Sensory gating and suppression of subjective peripheral sensations during voluntary muscle contraction. BMC Neurosci. 21:41. 10.1186/s12868-020-00592-2. 33003995 PMC7528260

[ref52] Tapia MC, Cohen LG, Starr A. 1987. Selectivity of attenuation (i.e., gating) of somatosensory potentials during voluntary movement in humans. Electroencephalogr Clin Neurophysiol. 68:226–230. 10.1016/0168-5597(87)90031-1. 2436883

[ref53] Tinazzi M et al. 1998. Effects of voluntary contraction on tibial nerve somatosensory evoked potentials: gating of specific cortical responses. Neurology. 50:1655–1661. 10.1212/wnl.50.6.1655. 9633707

[ref54] Wasaka T, Kakigi R. 2012. Conflict caused by visual feedback modulates activation in somatosensory areas during movement execution. NeuroImage. 59:1501–1507. 10.1016/j.neuroimage.2011.08.024. 21889595

[ref55] Wasaka T, Hoshiyama M, Nakata H, Nishihira Y, Kakigi R. 2003. Gating of somatosensory evoked magnetic fields during the preparatory period of self-initiated finger movement. NeuroImage. 20:1830–1838. 10.1016/S1053-8119(03)00442-7. 14642492

[ref56] Wasaka T et al. 2005a. Differential modulation in human primary and secondary somatosensory cortices during the preparatory period of self-initiated finger movement. Eur J Neurosci. 22:1239–1247. 10.1111/j.1460-9568.2005.04289.x. 16176367

[ref57] Wasaka T, Nakata H, Kida T, Kakigi R. 2005b. Changes in the centrifugal gating effect on somatosensory evoked potentials depending on the level of contractile force. Exp Brain Res. 166:118–125. 10.1007/s00221-005-2333-7. 15856201

[ref58] Wasaka T, Kida T, Kakigi R. 2012. Modulation of somatosensory evoked potentials during force generation and relaxation. Exp Brain Res. 219:227–233. 10.1007/s00221-012-3082-z. 22460200

[ref59] Wasaka T, Kida T, Kakigi R. 2017. Facilitation of information processing in the primary somatosensory area in the ball rotation task. Sci Rep. 7:15507. 10.1038/s41598-017-15775-x. 29138504 PMC5686197

[ref60] Wasaka T, Kida T, Kakigi R. 2021. Dexterous manual movement facilitates information processing in the primary somatosensory cortex: a magnetoencephalographic study. Eur J Neurosci. 54:4638–4648. 10.1111/ejn.15310. 33987876 PMC8361953

[ref61] Wikström H et al. 1996. Effects of interstimulus interval on somatosensory evoked magnetic fields (SEFs): a hypothesis concerning SEF generation at the primary sensorimotor cortex. Electroencephalogr Clin Neurophysiol. 100:479–487. 10.1016/s0921-884x(96)95688-x. 8980411

